# Assessment of the policy enabling environment for large-scale food fortification: A novel framework with an application to Kenya

**DOI:** 10.1371/journal.pgph.0003211

**Published:** 2024-05-16

**Authors:** Veronique Theriault, Lilian Kirimi, Ayala Wineman, Ephiphania Kinyumu, David Tschirley

**Affiliations:** 1 Department of Agricultural, Food, and Resource Economics, Michigan State University, East Lansing, Michigan, United States of America; 2 Tegemeo Institute of Agricultural Policy and Development, Egerton University, Nairobi, Kenya; PLOS: Public Library of Science, UNITED STATES

## Abstract

Large-scale food fortification (LSFF) programs have potential to improve a population’s nutritional status. Though their success depends heavily on the prevailing policy environment, few tools exist to understand this environment. To address this gap, we develop a novel framework to define and assess the policy enabling environment for LSFF. This easy-to-apply framework can be used in any setting to track progress and identify next steps for continued improvements. The policy enabling environment is conceptualized as having three domains—policy agenda setting, policy implementation, and policy monitoring and evaluation—each of which is captured through indicators that can be evaluated using existing documentation, key informant interviews, and/or a survey of stakeholder perceptions. To validate the framework and demonstrate how it can be operationalized, we apply it in Kenya, where a mandatory LSFF program for salt has been in place since 1978, and a program for packaged maize and wheat flours and vegetable oils was introduced in 2012. Per our assessment, Kenya has achieved the greatest success within the domain of policy agenda setting, has realized moderate success in policy implementation, and has a weaker record in policy monitoring and evaluation. The positive trajectory for many indicators points to a promising future for Kenya’s LSFF program. This assessment yields policy implications for Kenya to improve its policy environment for LSFF, especially around financial sustainability of the program; ways to improve the processes for surveillance and enforcement; efforts to support fortification among medium-and small-scale millers; and a need to improve the data landscape.

## 1. Introduction

Large-scale food fortification (LSFF) programs aim to ameliorate micronutrient deficiencies by improving the nutritional status of foods that are widely and frequently consumed in a population. Food fortification consists of “deliberately increasing the content of one or more micronutrients (i.e., vitamins and minerals) in a food or condiment to improve the nutritional quality of the food supply and provide a public health benefit with minimal risk to health” [1]. Food vehicles used in LSFF programs include wheat flour, maize flour, salt, sugar, rice, and edible oils and fats [2], and micronutrients added to food include vitamins A, B_2_, B_6_, and D, as well as folic acid, iodine, iron, and zinc [3, 4].

Food fortification programs are more successful in a policy environment that enables improvements in nutrition [4, 5]. Recent studies have found that mandatory food fortification programs are more successful than voluntary programs, leading to greater public health benefits [1]. Their success is mainly attributed to better monitoring and regulation. LSFF programs also work better when the targeted food sector is concentrated (i.e., small number of large formal food processing firms) [5], as enforcement costs for regulators are lower when fewer firms are involved. Large firms also have a greater capacity to absorb the costs associated with fortification and can scale their operations to keep costs down.

Other factors of relevance include tax policy. When LSFF programs rely on imported inputs, such as premix and equipment, import duties and taxes can dissuade firms from fortifying [5]. Studies also indicate that LSFF programs that go beyond the public and private sectors to build partnerships with other organizations (e.g., international and civil society organizations) tend to be better at promoting mutual accountability, since such organizations can provide support in advocacy, training, implementation, and regulation [4–6].

In the absence of a supportive policy environment, LSFF in many countries has not been able to reach its full potential [7]. As a result, micronutrient deficiencies remain a critical threat to public health. Yet, little is known about what makes a policy environment supportive to LSFF. There is a growing literature on the application of frameworks to evaluate LSFF programs [8, 9] but to our knowledge, no previous study has explicitly defined which policy elements matter and assessed their adequacy. We address this gap by developing a novel framework to assess the policy enabling environment (P2E) for LSFF.

The framework is comprehensive—spanning policy agenda setting, implementation, and monitoring and evaluation—and the method is straightforward and low-cost to implement. Each of its 18 indicators can be evaluated using existing documentation, key informant interviews, and/or a survey of stakeholder perceptions. Each indicator is scored and then summed to arrive at an overall measure that conveys whether the P2E is “marginally”, “moderately”, or “highly” favorable for LSFF activities. The framework can be applied to assess the P2E for a whole food fortification program or for a specific fortified food. It can also be applied at one point in time or periodically (e.g., to monitor and evaluate changes in the P2E for LSFF following an intervention/policy reform). The framework is applicable in any setting; however, its application is necessarily country-specific, and we expect different experiences to emerge as the framework is applied to different countries.

After introducing this novel framework, we apply it in Kenya, where an LSFF program was expanded about 10 years ago. We do this to demonstrate how the method can be used to understand the strengths and weaknesses of the P2E and glean specific policy recommendations to improve LSFF program effectiveness.

The remainder of the paper is organized as follows. Section 2 includes background on the concept of a “policy enabling environment” (P2E). Section 3 presents our novel framework of the P2E for LSFF. Section 4 includes background on the LSFF program in Kenya and presents an application of the framework to the Kenyan context. Section 5 concludes.

## 2. Background on the policy enabling environment

What, exactly, is a “policy enabling environment” (P2E) for LSFF? In the Food Fortification Global Mapping Study [10], enabling environment characteristics for different fortified foods, such as salt and vegetable oils and fats, are analyzed but the policy characteristics are not clearly defined. Although the role of the enabling environment for agricultural innovation [11–13] and business [14, 15] has been widely studied, we are not aware of a study that has defined or assessed the “policy enabling environment” for a particular program such as food fortification.

Four analytical frameworks influenced our thinking around how to assess the P2E for LSFF. First, Marketlinks [16] has developed a framework for the business enabling environment and uses it to assess the competitiveness of a value chain, understand how actors behave, and make predictions about how they will respond to different interventions. Second, the Kaleidoscope Model examines the drivers of policy change in general and discusses what factors shape the effectiveness of policy implementation [17]. Third, the PMCA (Policy inventory, Mapping of stakeholders, Constraint identification, and Actions) approach is used to analyze the policy system around agriculture, with a mapping of stakeholders to delineate their interests and influence on policy outcomes; identification of key constraints to policy reforms; and proposal of actions to remove these constraints [18]. The fourth framework we reference is the Women’s Empowerment in Agriculture Index (WEAI), which measures women’s empowerment at the project level to discern whether project interventions are effective in empowering women [19].

These frameworks share several elements that we incorporate in our framework. First, most contain a feedback loop to capture the dynamic nature of a policy/business environment. For instance, major events, such as the Global Summit on Food Fortification in 2015, may have a lasting effect on policy design and may shift the position of different actors toward food fortification, necessitating the inclusion of a feedback loop to capture these effects. Second, each divides the policy process into domains, such as the identification of a policy priority, the intervention/policy implementation, and policy or program monitoring and evaluation. Third, the empirical application of these frameworks often entails stakeholder mapping. Fourth, most frameworks include a set of indicators to characterize each domain or measure the policy/intervention outcomes. Toward this end, information is drawn from secondary data, key informant interviews, focus groups, and/or surveys.

As the “policy enabling environment” for LSFF has not been mapped previously, a new framework is needed. We incorporate each of the aforementioned features in a novel framework designed for this purpose.

## 3. Policy enabling environment for LSFF

### 3.1 Framework

In this study, we understand the policy enabling environment (P2E) for LSFF to be the whole policy landscape that influences and enables or disables fortification activities. This landscape encompasses formal elements (such as laws and regulations, trade agreements, and public infrastructure), and informal elements (such as cultural and social norms) that can facilitate or hinder food fortification.

**Fig 1:**
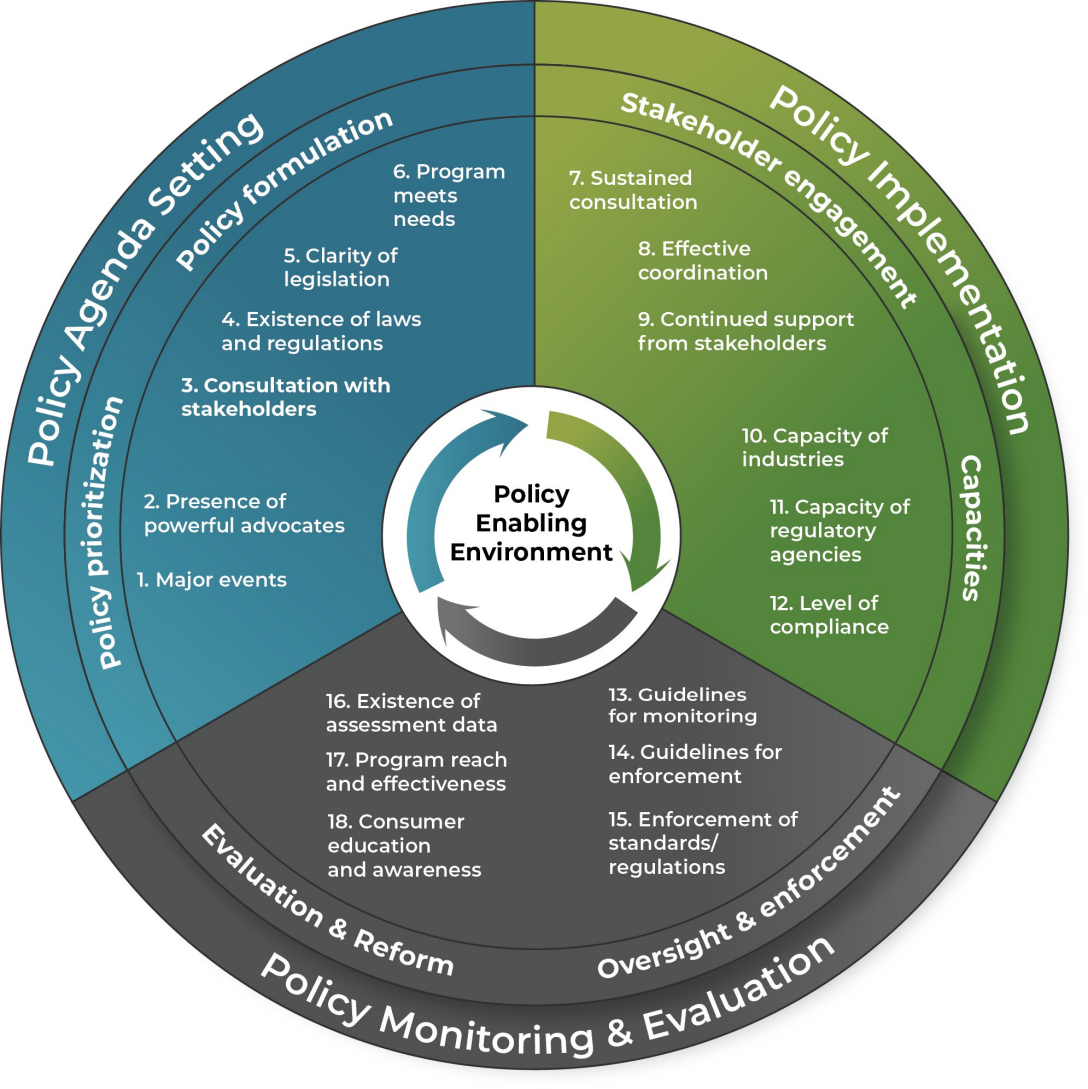
Framework of the policy enabling environment for large-scale food fortification.

We conceptualize three domains in the P2E: (1) policy agenda setting; (2) policy implementation; and (3) policy monitoring and evaluation. In an enabling environment, each domain must be strong and must reinforce the others. In Fig 1, the outer circle represents these three domains.

Each domain contains two elements (middle circle), and these elements are measured using two or more indicators for each (inner circle). The framework is circular to convey interconnections in the environment, with arrows in the center that indicate a mutually reinforcing and iterative process. Using multiple indicators allows for measures that more precisely capture the multiple dimensions of each domain [20]. The set of indicators was selected based on a review of the relevant literature and with the purpose of minimizing the risks of omission of relevant indicators, overrepresentation of some elements, and inclusion of irrelevant indicators [21]. The set of indicators was further reviewed and validated in the course of applying this framework to Kenya, as discussed in section 4. Only the validated set of indicators is presented. We include 6 indicators under each domain for a total of 18 indicators; these are defined in Table 1.

**Table 1:** Description of indicators.

Domains	Elements	Indicators	Description
Policy agenda setting	Policy prioritization	1. Major events	A major event has attracted the attention of the public/industry/policy makers to LSFF.
		2. Presence of powerful advocates	There are powerful advocates for LSFF in the country.
	Policy formulation	3. Consultation with stakeholders	There was consultation among stakeholders in the design of the LSFF legislation.
		4. Existence of laws and regulations	There exist laws and/or regulations on LSFF.
		5. Clarity of legislation	The legislation related to LSFF is clear/easy to understand.
		6. Program meets needs	The LSFF program is designed, based on evidence, to meet the population’s needs in terms of types and amounts of nutrients and choice of food vehicle.
Policy implementation	Stakeholder engagement	7. Sustained consultation	There is sustained consultation among stakeholders in the implementation of the LSFF program (i.e., the program is well communicated and understood).
		8. Effective coordination	There is effective coordination among stakeholders in the implementation of the LSFF program (i.e., roles and responsibilities are well defined and complementary).
		9. Continued support from stakeholders	There is continued support in terms of enthusiasm, engagement, and assistance from stakeholders in the implementation of the LSFF program.
	Capacities	10. Capacity of industries	Industries have adequate financial/human/physical capacity to meet the fortification requirements.
		11. Capacity of regulatory agencies	Regulatory agencies have adequate financial/human/physical capacity to monitor and enforce the fortification requirements.
		12. Level of compliance	There is a satisfactory level of industry compliance with the fortification requirements.
Policy monitoring and evaluation	Oversight and enforcement	13. Guidelines for monitoring	There exist clear guidelines for monitoring LSFF.
		14. Guidelines for enforcement	There exist clear guidelines for enforcement of LSFF.
		15. Enforcement of standards/regulations	The fortification requirements are adequately enforced (i.e., they are enforced consistently, fairly, and transparently).
	Evaluation and reform	16. Existence of assessment data	Data on LSFF (e.g., volumes, compliance rates) and population micronutrient deficiencies are tracked and reported over time.
		17. Program reach and effectiveness	Program reach and effectiveness is satisfactory.
		18. Consumer education and awareness	Consumers are aware of the importance of fortified foods, accept fortified foods, and know how to identify fortified products in the market.

Source: Authors

The first domain, policy agenda setting, involves identifying the issue of micronutrient deficiencies as a priority, placing LSFF on the formal policy agenda (policy prioritization), and formulating policy. Many issues may be considered important, but just a few make it to the policy agenda. Following Resnick et al. [17], we assess the prioritization of food fortification based on two indicators: the country’s participation in a major event, such as the Global Summits on Food Fortification, which can attract the attention of the public, food industries, and/or policy makers (indicator 1), and the presence of powerful advocates pushing for action (indicator 2).

Policy formulation refers to the selection, development, and legitimation of policy instruments/actions for LSFF, including the drafting and adoption of laws and regulations. Indicator 3 captures whether there was consultation among stakeholders in the design of the food fortification legislation. As highlighted by Cairney [22], consultation is critical to ensure that the policy is accepted. Indicators 4 and 5 capture whether food fortification laws and regulations exist and whether these are clear and easily understood by stakeholders. The existence of codified policies, such as a mandate, influences the success of a fortification program [1]. Finally, policy instruments must address a recognized need (indicator 6). That is, the fortification program should be designed based on evidence in order to meet the population’s needs in terms of types and amounts of nutrients and choice of food vehicles to address micronutrient deficiencies and ensure food safety and quality [10].

Identifying food fortification as a priority and adopting relevant policies are necessary but not sufficient to create an enabling policy environment. Policy implementation encompasses the activities that put laws and regulations into effect. Indicator 7 measures whether there is sustained consultation among stakeholders during implementation to ensure that the program is well communicated and understood, even after the initial design stage. This is important as successful implementation rests on having a well-understood policy [22]. Indicator 8 captures whether there is effective coordination among stakeholders through well-defined and complementary roles and responsibilities and mechanisms for needed information exchange and dialogue. Indicator 9 assesses whether there is continued support in terms of enthusiasm, engagement, and assistance from stakeholders in implementing the program.

In addition, there must be adequate financial, human, and physical capacity of the food industries to comply with fortification policies (indicator 10), as well as adequate financial, human, and physical capacity of the regulatory agencies to monitor, control, and enforce product quality and safety (indicator 11). Successful implementation is also reflected in satisfactory compliance by food companies with the fortification requirements (indicator 12).

The last domain, policy monitoring and evaluation, refers to monitoring compliance with and enforcing existing laws and regulations and evaluating and reforming the policy, if necessary. Effective monitoring is needed to identify gaps in implementation. This is captured through the existence of clear guidelines for monitoring (indicator 13). Program success also requires the existence of clear guidelines for enforcement (indicator 14), along with the enforcement of standards and regulations in a manner that is consistent, fair, and transparent (indicator 15).

An enabling policy environment also includes evaluation to assess whether the policy is achieving the desired outcomes in order to reform the policy agenda or implementation, as needed. This requires regular tracking and reporting of assessment data, such as production and sales volumes and rates of compliance (indicator 16) [10]. In addition, adequate efforts must be made to evaluate, through empirical evidence, the reach and effectiveness of the program (e.g., by measuring rates of compliance, assessing the availability and affordability of fortified products in local markets, and determining the impacts on public health) (indicator 17). Finally, a strong enabling policy environment requires consumers who are aware of the importance of fortified foods, accept fortified foods, and know how to identify them in the market, especially when there is no nationwide mandate (indicator 18). The indicator applies to both mandatory and voluntary fortification, since consumers who disapprove of fortified foods can opt out of purchasing and consuming them.

### 3.2 Data collection and evaluation

As most indicators are qualitative, we measure them through the lens of key stakeholders’ opinions. Eliciting expert opinions is especially useful when information and data are sparse; gathering the opinions of a wide range of experts in different positions within the sector allows us to synthesize the limited available knowledge and avoid bias from one type of expert influencing the assessment [23]. Different methods exist to elicit stakeholder and expert opinions, including individual interviews, expert group discussions, semi-structured questionnaires administered in person or online, or a combination thereof. To keep the process simple and low-cost, we suggest two methods for gathering experts’ views: individual interviews and an online survey. Individual interviews are more time-consuming but tend to be less financially demanding than expert group elicitation [23].

We developed a semi-structured interview guide for the individual interviews with questions about each domain, element, and indicator of the framework (see Fig 1 and S1 Text). The questions probe for overall opinions and also seek detailed information on each indicator. There is no “magic number” of experts to interview. In their application of the Kaleidoscope Model to the topic of vitamin A fortification in Zambia, Resnick et al. [17] conducted semi-structured interviews on policy reform episodes with representatives of 19 institutions. Sitko et al. [18] recommend that key informant interviews be conducted with at least one stakeholder in the public, private, and civil society sectors to ensure a minimum level of representativeness at each stage of the PMCA approach. As previous work indicates that little information is generated after interviewing 20 experts on a specific topic in most qualitative research [24, 25], we suggest about 20 expert interviews, while keeping in mind that interviews should continue until there is data saturation—that is, until no new insights are obtained from an additional interview.

In addition, we designed an online survey to elicit stakeholders’ perceptions of the P2E for food fortification (see S2 Text). The survey comprises a set of statements of relevance to the domains, elements, and indicators of the framework. Respondents indicate the extent to which they agree with each statement; they also have an opportunity to provide additional comments. The question of sample size for this survey is not straightforward. The number of participants in surveys is usually greater than for individual interviews. As we aim to capture opinions on a relatively narrow topic, we suggest a target of 40 stakeholders to complete the survey.

To identify experts who can inform the assessment of the P2E for LSFF in a country, it is helpful to map the value chain for food fortification (see Fig A in S3 Text). This entails identifying individuals, firms, organizations, and agencies involved in food procurement, processing, and trade, as well as those involved in support services (development partners, government agencies at all relevant levels, researchers, and civil society organizations). A stakeholder list can be generated through an online search and/or a snowball approach in which stakeholders are asked to provide additional contacts that can be approached. From this list, individuals can be selected for interviews and/or invited to participate in the survey. As a last step, a validation workshop should be organized with key stakeholders to review, discuss, and validate the results from the application of the framework.

### 3.3 Calculation of the index

Information gleaned from existing documentation, key informant interviews, and the stakeholder perceptions survey can be triangulated to arrive at an understanding of each indicator within the LSSF framework (Fig 1). To construct an index of this environment, information from all three sources is assessed, and a four-point Likert scale is used to score each indicator. With reference to the descriptions of the indicators provided in Table 1, the Likert scale is as follows: 1 = completely disagree; 2 = somewhat disagree; 3 = somewhat agree; 4 = completely agree with the description of the indicator (i.e., the indicator is satisfied). We omit the option “neither disagree nor agree” to force a decision.

The values for each indicator are summed up, with a minimum overall score of 18 and a maximum of 72. Note that each indicator is given equal weight, and there are an equal number of indicators for each of the three domains. The P2E is considered “highly favorable” to large-scale food fortification if the summed score is over 54, “moderately favorable” if it is between 36 and 54, and “marginally favorable” if it is less than 36. These thresholds were calculated by dividing the possible range of scores equally into three groups. The index can be computed for a country’s LSFF program in its entirety. However, in countries with multiple food vehicles or sub-programs, the index can also be computed separately for each commodity.

### 3.4 Ethics statement

This study has been reviewed and determined to be exempt by the Michigan State University Institutional Review Board. Ethical approval was provided by the Egerton University Ethics Review Committee (EUERC). Research license was provided by the National Commission for Science, Technology, and Innovation (NACOSTI) in Kenya.

At the start of the interview, a research consent statement was read to each key informant, and by continuing with the interview, they indicated their voluntary consent to participate in this study. The first page of the stakeholder perception survey was a research consent statement, and by continuing with the survey, the stakeholder indicated their voluntary consent to participate in this study.

### 3.5 Inclusivity in global research

Additional information regarding the ethical, cultural, and scientific considerations specific to inclusivity in global research is included in the S4 Text.

## 4. An application to Kenya

### 4.1 Background on LSFF in Kenya

National food fortification requirements were first introduced in Kenya in 1978 when the Iodine Deficiency Disorder legislation made it mandatory for salt to be fortified with iodine [26]. Large-scale food fortification, as highlighted in the National Food and Nutrition Security Policy [27] and the Kenya National Nutrition Action Plan (2018–2022) [28], has since been embraced as a key intervention to enhance micronutrient intake, and mandatory fortification of vegetables oils and wheat and maize flours was enacted in 2012 (What is commonly referred to as maize meal in Kenya is considered by the worldwide milling industry to be flour). In 2015, the standards for oils and flours were made explicit within Kenyan policy [29]; the standards are presented in Table A in S3 Text. As stated in the legislation, these standards apply only to packaged oils, wheat flour, and dry milled maize products, regardless of the size of the food processing firm [26].

In Kenya, packaged maize flour is largely associated with that produced by roller mills and not the much smaller hammer (posho) mills [30]. Hammer mills use simpler, smaller-scale technology, with many of the smallest hammer mills operating as toll mills (fee-for-service mills) that process grain brought to the mill by customers. There are several reasons why the law targets only packaged products: (1) It would be logistically and technically more demanding to monitor compliance in the country’s numerous hammer mills; (2) the incremental costs of fortification are higher for smaller scale millers, as there are economies of scale in fortification; and (3) size-appropriate fortification technologies (e.g., dosers) are difficult to access [31–33]. Many governments and development partners focus their food fortification initiatives on large firms, which are sometimes delineated as those with a processing capacity of more than 50 metric tons per day [6, 31, 32, 34]. Nevertheless, the Government of Kenya has expressed an intention to support fortification by small- and medium-scale industries [29].

Food fortification in Kenya is coordinated by the Ministry of Health, Nutrition and Dietetics Unit (MoH-NDU), and premix suppliers and distributors are certified by the Kenya Bureau of Standards (KEBS) and registered annually by the MoH-NDU [29]. The certification process involves an application, an assessment (including inspection of the production facility and product sampling), and an evaluation (laboratory analysis of the samples) [35]. A fortification logo, developed in 2006, is placed on the package of fortified foods. Surveillance of fortification is conducted by the Food Safety Unit, the National Public Health Laboratory, and County Governments [29]. The extent of surveillance varies across counties, contingent on resource availability.

Though food fortification is officially coordinated by the Ministry of Health, the Kenya National Food Fortification Alliance (KNFFA) oversees fortification activities [29]. The KNFFA was established in 2005 and brings together various public and private sector agencies and development partners. Membership includes the Ministry of Health; KEBS; industry representatives; the Ministries of Industrialization and Trade; and development partners [26].

### 4.2 Data and methods

Semi-structured interviews with key informants were conducted virtually from June to September 2022. In total, 19 interviews were conducted with representatives of government (at the national and county levels); industry; civil society organizations; development partners; and academia. Two interviews included two informants from the same office, bringing the number of key informants to 21 (See Table B in S3 Text). In addition to the key informant interviews, a short stakeholder perceptions survey was conducted to gauge the extent to which stakeholders perceive the LSFF program to be functioning well and achieving its goals. Participants were identified through online research and through a snowball method of asking key informants and other contacts to identify additional individuals who are knowledgeable about LSFF. The survey was administered online, and invitations to participate were sent between July and September 2022. In total, 46 stakeholders completed the survey out of 60 who received an invitation (see Table C in S3 Text). It should be emphasized that this is not a representative sample of LSFF stakeholders in Kenya; rather, the survey can only measure sentiments among those who opted to complete the survey. All experts who participated in the interviews were invited to a validation workshop, held in Nairobi, Kenya in October 2022. Of the 21 experts who were invited, 13 attended this workshop.

### 4.3 Results

Based on information gleaned in the interviews, survey, and existing documentation, we assessed the extent to which the 18 indicators of the framework (Fig 1) are found in the P2E for the LSFF program in Kenya. (Survey results are available in Figs B-D in S3 Text) The values for each indicator (with 1 indicating that we “completely disagreed” with the descriptive statement in Table 1, and 4 indicating that we “completely agreed”) are presented in Table 2. Per our assessment, Kenya has achieved the greatest success in policy agenda setting, has obtained moderate success in policy implementation, and has a weaker record in policy monitoring and evaluation. Summing the values across the 18 indicators, this translates into a score of 49—a “moderately favorable” P2E.

**Table 2:** Achievement of LSFF policy enabling environment indicators in Kenya.

Domains	Elements	Indicators	Stakeholder perception survey: Somewhat or completely agree (%)	To what extent does Kenya achieve this indicator? (1 to 4)^a^
Policy agenda setting	Policy prioritization	Major events	79	4
		Presence of powerful advocates	80	4
	Policy formulation	Consultation with stakeholders	n/a	4
		Existence of laws and regulations	n/a	4
		Clarity of legislation	78	4
		Program meets needs^c^	80	4
Policy implementation	Stakeholder engagement	Sustained consultation	70	4
		Effective coordination^c^	38	2
		Continued support from stakeholders^b^	55	3
	Capacities	Capacity of industries^c^	36	2
		Capacity of regulatory agencies^c^	41	2
		Level of compliance	46	2
Policy monitoring and evaluation	Oversight and enforcement	Guidelines for monitoring	---^b^	2
		Guidelines for enforcement	---^b^	2
		Enforcement of standards/regulations	41	2
	Evaluation and reform	Existence of assessment data	26	1
		Program reach and effectiveness	32	2
		Consumer education and awareness^c^	23	1
				**TOTAL = 49**

^a^ Scale of 1 to 4, with reference to the descriptions/statements in Table 1: 1 = completely disagree, 2 = somewhat disagree, 3 = somewhat agree, 4 = completely agree

^b^ This indicator was not initially reflected in the survey as administered in Kenya, though it is reflected in the questionnaire offered in S2 Text.

^c^ This is an average score.

#### 4.3.1 Domain I: Policy agenda-setting

*Participation in major events*. Kenya has participated in or hosted multiple large events around the topic of LSFF, earning a value of 4 for this indicator. Seventy-nine percent of respondents in the stakeholder perceptions survey agreed (by indicating that they either “completely” or “somewhat” agreed) that a major event has attracted the attention of the public, industry, and/or policy makers to LSFF, and one key informant noted that they “have been seeing a fortification event every year or so.” For example, a Kenya National Food Fortification Summit was held in June 2021 and was perceived as quite successful. These summits are an opportunity for stakeholders to learn about the country’s progress, share experiences, brainstorm solutions to various challenges and build awareness of advances in LSFF in other parts of the world.

*Presence of powerful advocates*. Almost 80% of survey respondents agreed that there are powerful advocates for LSFF in the country. This earns Kenya a value of 4 for this indicator. At the national level, the Ministry of Health, Division of Nutrition and Dietetics has been the main champion of LSFF, and it is supported in compliance and enforcement by KEBS (in the Ministry of Industrialization and Trade). Champions also include processors complying with food fortification regulations and standards. Some millers even adopted fortification before it became mandatory, and the Cereal Millers Association has played a key role in the KNFFA from its inception.

*Consultation with stakeholders in policy design*. Stakeholders generally felt quite positive regarding the extent of consultation in the initial design of LSFF legislation, earning Kenya a value of 4 for this indicator. From the start, it seems a diverse set of stakeholders were engaged in developing the fortification standards. In fact, we heard that the introduction of mandatory standards was partly motivated by industry, as firms that were fortifying in accordance with non-mandatory recommendations wanted these to be required of their competitors to foster a level playing field. The KNFFA’s leadership comes from the private sector, while government officials comprise the secretariat. According to key informants, when the LSFF mandate was first extended to maize and wheat flours and edible oils in 2012, there was “a large convening” inclusive of industries, research/training institutes, various arms of government, and at least one consumer outreach organization, among others. However, key informant interviews did reveal certain oversights in stakeholder engagement during policy agenda setting. Specifically, industry engagement in the initial stages was limited to associations representing large-scale firms. The legal framework was already in place before small- and medium-scale millers had formed their associations or organized their involvement in LSFF.

*Existence of laws and regulations*. As noted earlier, Kenya has had a national fortification requirement for salt since 1978, and for vegetable oils and maize and wheat flours since 2012. The latter is found in an amendment to the Food, Drugs and Chemical Substances Act of the Laws of Kenya CAP 254, Notice No. 62 [29]. The law was reviewed in 2015 to harmonize Kenyan standards with those of the East African Community, and Kenyan standards for oils and flours were made explicit in CAP 254, Notice No. 157 [29]. Guidelines for premix imports are laid out in Premix Requirements KS 2571, and the national standards for premix have been reviewed as recently as 2020. The existence of these laws and regulations earns Kenya a value of 4 for this indicator.

*Clarity of legislation*. Stakeholders generally felt positive regarding the clarity of the legislation, with 78% of survey respondents agreeing on this point. Overall, the legislation is clear on which processors are required to fortify which products, with which fortificants, and at what level. This earns Kenya a value of 4 for this indicator, even as we can identify room for improvement. The Food, Drugs, and Chemical Substances Act (in which food fortification is anchored) identifies public health officers (PHOs) as the implementers of the program, and their mandate is considered to be clear. However, the roles of different stakeholders are defined in various program-level documents (e.g., strategy or guidance documents) produced by the KNFFA, and not in the law itself. While the roles of various players are not spelled out precisely in the legislation, this does not seem to be a major source of confusion for stakeholders. There are, however, two areas where clarity can be improved. First, according to informants, the food fortification law does not specify penalties for noncompliance. Second, how “compliance” is measured in a context where the mandate only applies to processors that produce packaged products is unclear. These two areas are further discussed below.

*Program meets needs*. Informants largely approved of the LSFF program structure, and 85% of survey respondents agreed that the choice of several widely consumed staple foods as food vehicles is appropriate. Some of the products, such as oil, wheat flour, and salt, are especially suitable for fortification owing to the industry structure, as almost all production in the country is large-scale. Eighty-two percent of survey respondents agreed that the mandated types and amounts of nutrients are appropriate, and 72% agreed with the statement, “Overall, the large-scale food fortification program is well-designed to meet the population’s needs in terms of fortified food availability, affordability, and quality.” With little equivocation heard among informants, this earns Kenya a value of 4 for this indicator.

#### 4.3.2 Domain II: Policy implementation

*Sustained consultation*. Seventy percent of survey respondents agreed that “there is consultation and coordination among stakeholders in the implementation of the LSFF program.” The key informant interviews revealed general satisfaction with the extent and quality of consultation and communication, earning Kenya a value of 4 for this indicator. The KNFFA’s mandate is to spearhead the planning, implementation, and monitoring of fortification initiatives and to guide public-private sector coordination. Initially, large-scale millers served as the chair for the KNFFA. Now, the chair position is assumed by medium- and small-scale millers. The KNFFA is widely considered to be effective, with regular meetings and frequent sharing of experiences, ideas, and information. While the milling industries, inclusive of premix suppliers, are active in these meetings, it was noted that the salt and oil industries are not as engaged. We did hear that consultation is most successful at the national level, and several informants felt that the sort of consultation that occurs via the KNFFA ought to be cascaded to the county level. Nairobi County has formed a county multi-sectorial food safety coordination committee. The Nairobi County Fortification Alliance is regarded as successful and inclusive, though this success is not mirrored in all counties.

*Effective coordination*. Stakeholders were less confident about the effectiveness of coordination in the implementation of the LSFF program. Note that this distinction between consultation and coordination was not initially reflected in the survey as administered in Kenya, though it is reflected in the questionnaire offered in S2 Text. Forty-one percent of respondents felt that there is adequate coordination of monitoring among stakeholders, while 35% felt there is adequate trust among stakeholders. Kenya earns a value of 2 for this indicator. We did hear of numerous instances of coordination and purposeful collaboration amongst the stakeholders. For example, counties receive support for monitoring/surveillance from the Divisions of Nutrition and Food Safety at the national level. Nevertheless, we also heard of obstacles to coordination, especially among different levels of government. Food fortification in Kenya is a devolved function, and the 47 counties are responsible for inspections. Since devolution, the Division of Food Safety must communicate with the counties through the Council of Governors. This channel of communication can sometimes be tedious and bureaucratic, with extended delays before a response is received.

*Continued support from stakeholders*. A total of 48% and 61% of respondents agreed that there are adequate public and private investments, respectively, in food fortification. Kenya earns a 3 for this indicator. Overall, the government and other players remain proud of the LSFF program; this sentiment was displayed in almost all interviews. Furthermore, we heard of efforts made by various stakeholders to support the program. To incentivize leadership within industry, organizers of the Food Fortification Summit presented trophies, gifts, and other tokens of appreciation for companies and other players that have been excelling in their food fortification activities. At the same time, there was some variation in enthusiasm. For example, while the milling industries are active in KNFFA meetings, the salt and oil industries attend less frequently. The associations for different food vehicles and firm types also vary in their commitment. They tend to support fortification only through training and the provision of information on different premix suppliers. Additionally, there is variation in county attention to LSFF. We heard that five counties (Nakuru, Mombasa, Nairobi, Kiambu, and Turkana) have so far demonstrated a notable commitment to fortification, while support is lower elsewhere.

*Capacity of industries*. The capacity of processors to comply with the fortification mandate varies with the food product and firm size. For this reason, it is difficult to assign a single value to capture the overall capacity of relevant industries in Kenya. In total, 28%, 42%, and 39% of survey respondents felt that industry actors have adequate financial, human, and physical capacities, respectively, to meet the fortification requirements. Kenya earns a value of 2 for this indicator.

There are several reasons why large-scale firms have a greater capacity to engage in fortification. They are likely to already have in place a system of quality control with logbooks, a quality assurance manager, and perhaps their own laboratory to test the quality of premix. They also already have financial resources and purchase patterns in place such that the additional requirement to procure premix (which is imported) is less of a burden. Most large-scale firms also have a brand that they do not want to see tarnished; if they are caught not complying with the fortification mandate, their brand may be pulled from the shops. In contrast, it is more difficult for small/medium-scale firms to absorb the cost of fortification. They do not have personnel whose primary responsibility is quality assurance; they lack the capacity to install micro-dosers and conduct internal monitoring; and without their own laboratory facilities, they can only trust that the premix they procure is of high quality. These millers also face a mismatch in terms of their scale of production and the large units in which premix is sold.

The difference in capacity between large- and smaller-scale firms translates into variation in capacity across different food vehicles. This is because the industry structures for salt, vegetable oils, wheat flour, and maize flour are so heterogeneous. Salt is by far the most concentrated industry with three large-scale processors in Kenya. The vegetable oils and wheat flour industries are also relatively concentrated and are dominated by medium- or large-scale companies. However, the maize flour industry is far less concentrated, with numerous medium- and small-scale mills. It follows that industry capacity is lower for maize flour.

*Capacity of regulatory agencies*. Regulatory agencies lack human, physical, and financial capacity to surveil and enforce the LSFF program. Only 35% of survey respondents felt that regulatory agencies have adequate capacity to monitor fortification activities, and 46% felt they have adequate capacity to enforce the fortification requirements. Kenya earns a value of 2 for this indicator. Training and stakeholder meetings on food fortification have taken place at the national level and at the level of county management; however, knowledge has not been disseminated to the subcounty levels and to technicians. There are about 21 (out of 47) counties with County Food Safety and Fortification Coordination Committees. However, these committees are sometimes in flux; they can be dissolved or reconstituted with each election. A lack of local government commitment to fortification can undermine the capacity of those tasked with monitoring and enforcement.

Kenya relies heavily on its development partners to finance LSFF activities, and it was unclear whether government would be able to sustain the activities without donor support. With budget limitations, KEBS is unable to conduct impromptu visits to food processors to take samples for analysis. This limits the effectiveness of industry surveillance. The surveillance at market level is particularly weak due to financial constraints at the county level; regulators do not have the financial capacity to carry out their mandate. Regulatory authorities also lack laboratory capacity. All (or almost all) samples collected throughout the country are sent to Nairobi for analysis, and only KEBS is able to analyze samples to determine compliance. The National Public Health Laboratory can be slow in turning around the samples due to lack of funds for reagents and equipment, and while they are able to test for zinc and iron, we learned that they must send the samples elsewhere to test for vitamin A.

*Level of compliance*. In the stakeholder perceptions survey, 46% of respondents agreed that there is satisfactory compliance with the fortification requirements. Compliance rates vary widely across food vehicles and firm sizes. Kenya earns a value of 2 for this indicator. Overall, however, compliance is on a positive trajectory. For example, overall compliance increased from 16% and 27% in 2017 to 28% and 35% in 2020 for maize flour and wheat flour, respectively, and compliance at the industry level (i.e., the percent of brands fortifying) was 46% and 84% for maize flour and wheat flour, respectively [36]. Not surprisingly, we also heard that compliance varies with the size of maize flour processors, with lower compliance among small and medium-scale processors. Although nearly all salt is fortified, as of 2014, about two-fifths of salt samples in a market surveillance study were compliant with the national standards [29].

Two caveats accompany the compliance indicator. First, as with several other indicators, it is difficult to assign one value to the whole LSFF program, as compliance varies across food products and firm sizes. Second, it is often unclear whether the measure of adherence to the fortification requirements include (or should include) firms that are not legally required to follow the mandate. It is often unclear whether each measure of compliance referred to the share of firms that fortify, the share of quantity sold on the market that is fortified, the share of households that consume fortified products, or something else.

#### 4.3.3 Domain III: Policy monitoring and evaluation

*Guidelines for monitoring*. Guidelines for monitoring the LSFF program in Kenya exist, as in the Monitoring Guideline KS 2765 [37] and the National Monitoring and Evaluation Framework [35], which accompanies the National Food Fortification Strategic Plan [29]. However, Kenya earns a value of 2 for this indicator due to their vagueness and limited availability. We learned that technical manuals and protocols for regulatory monitoring were first developed at the regional level by the East, Central and South Africa Health Community (ECSA-HC) with support from development partners. However, we also heard that these manuals are not comprehensive but rather general tools. Some counties, such as Nairobi, have formulated their own guidelines for monitoring. However, the availability of local guidelines is highly variable.

*Guidelines for enforcement*. There seem to be limited guidelines for enforcement of the fortification mandate, earning Kenya a value of 2 for this indicator. According to informants, the food fortification law (CAP 254) does not specify penalties for noncompliance. In fact, penalties for noncompliance are not specified in any document; rather, they are left to the discretion of local officials. If a firm is not compliant, the local prosecutor typically discusses each case with the court (i.e., the magistrate), deciding on a penalty that seems to fit the magnitude of the problem. In response to this, Nairobi County prepared a Food Safety and Fortification Bill in 2022 which contains a specific penalty for noncompliance.

*Enforcement of standards and regulations*. Forty-one percent of survey respondents felt the fortification requirements are adequately enforced. However, information gleaned in the key informant interviews suggests that, in some key areas, Kenya is failing when it comes to the enforcement of standards and regulations. Kenya earns a value of 2 for this indicator.

Multiple informants expressed the view that the regulatory structure is disjointed, with KEBS and county health department personnel working in isolation. KEBS is headquartered in Nairobi while the public health officers (PHOs) are based at the county level, and interaction occurs only at infrequent meetings. The PHOs are responsible for sampling food products in markets, while KEBS is responsible for monitoring among firms. Nevertheless, we heard fuzzier narratives about the responsibilities of each entity, with some county-level public health departments saying that they also visit maize mills, while KEBS also works in markets.

There are two additional causes for concern around the enforcement of fortification standards. First, counties may be strong in enforcing fortification requirements among processors operating within the county, while they are weak in controlling the fortification status of products that come from elsewhere to be sold within the county. In such a case, local processors feel they face unfair competition. Second, the lack of codified penalties for noncompliance with the fortification mandate means that prosecutors have great discretion in enforcement. Capacity building for prosecutors is needed to drive more consistent enforcement across counties.

*Existence of assessment data*. Kenya falls short in the collection of data on LSFF activities and impacts, and just 26% of survey respondents agreed that data on food fortification are tracked and reported consistently. For this reason, Kenya earns a value of 1 for this indicator. The Ministry of Health initially set up an online platform through which industries would be required to report the amount of premix imported and/or used and the amount of fortified products produced. The intention of this portal was to continuously estimate compliance with the fortification mandate. Any discrepancy between the amount of premix procured and the amount that should have been used might be discernible in the industry data portal—if it were functional. However, it is not functional, and the database is now being moved to the Ministry of Health website. This transition has been slow; at the time of this study, the portal at the MoH site was still not functional. Its absence precludes triangulation of other measures of compliance with the fortification mandate. Moreover, the last national survey on micronutrient consumption was conducted in 2011 [38]. Without updated national statistics, it is challenging to evaluate the impacts of the fortification program on rates of micronutrient deficiency.

*Program reach and effectiveness*. Approximately one-third of stakeholders agreed that there are adequate efforts to evaluate the impact and effectiveness of the LSFF program. Many feel positive about the future trajectory of the LSFF program, although additional efforts are needed to reach more consumers. Acknowledging the limited data to ascertain the program reach and effectiveness, Kenya earns a value of 2 for this indicator.

Regarding the program’s impacts on public health, there is some promising evidence related to iodine deficiency, which causes goiter. Due in part to the long-established salt fortification mandate, the rate of iron deficiency among children under 5 years of age has declined from 73% in 1999 to 13% in 2011 [29]. Although many challenges are recounted around fortification by small-scale maize millers, we heard that in recent years, smaller millers have also wanted to be counted as contributing to the “Big Four Agenda”, former President Kenyatta’s overarching plan for Kenya’s betterment [39]. Nevertheless, the large share of maize flour that is not fortified, especially from posho mills, indicates that Kenya has considerable room for improvement. Though evidence of impact is limited and challenges in the program persist, informants’ confidence in the LSFF program’s effectiveness suggests that Kenya is on its way to achieving this indicator.

*Consumer education and awareness*. Consumers in Kenya overwhelmingly lack awareness of, or appreciation, for fortified foods. Just 27% of survey respondents agreed that consumers know how to identify fortified products on the market, and 19% felt that consumers are aware of the importance of fortified foods. For this reason, Kenya earns a value of 1 for this indicator. Civil society organizations lack the resources and human capacity to raise awareness of fortification. It follows that consumers are not well informed of fortification activities, and they purchase products based almost entirely on price.

### 4.4 Policy implications for Kenya

This assessment of the P2E for LSFF in Kenya indicates that Kenya has a “moderately favorable” P2E. We frequently heard that Kenya has improved over time in indicators found in each of the domains of the P2E framework. This positive trajectory points to a promising future for Kenya’s LSFF program.

Our assessment of the P2E for LSFF in Kenya yields several important policy implications. First, financial sustainability is a persistent challenge, a pattern also documented elsewhere [40]. There is a need for both national and county governments to commit resources to undertake LSFF activities, to establish the necessary institutional structures, and to sustainably build capacity for their surveillance teams.

Second, effort should be focused on improving surveillance and enforcement. This recommendation is reflected in other reviews of LSFF programs [6, 40, 41]. More training is required to ensure the relevant actors have the capacity to monitor adequately in their jurisdiction. Impromptu visits, not only pre-scheduled ones, should be conducted. Beyond verifying that fortification equipment is installed and functioning, these visits should ascertain that premix is stored under proper conditions to maintain its quality. There is a need to ensure the quality of premix sold in Kenya by testing it for all fortificants and also by ensuring that licensed, high-quality premix suppliers can be identified easily.

Third, data around LSFF in Kenya needs improvement—a pattern also observed in other countries [42]. This yields several specific policy prescriptions: There is a need to reduce the turnaround time for testing samples to ensure the accuracy and usefulness of the results. This may be achieved by devolving the testing function to other satellite laboratories, helping counties to set up new laboratories, and guiding counties to organize their laboratory needs through regional blocs. The data portal for industry reporting should be relaunched to ensure that there is some scope for triangulating measures of compliance, and a new round of the Kenya National Micronutrient Survey should be conducted soon. Moreover, a culture of data utilization needs to be cultivated, something that could be promoted through reference to data at Kenya’s periodic summits on food fortification.

Fourth, the definition of “compliance” should be clarified. This will help stakeholders understand what is being measured when a given measure of compliance is reported. If some firms are not required to adhere to the fortification mandate, then the measure of “compliance” should be limited to those firms that do face a legal mandate, while another term (such as “coverage” or “participation”) might be used to capture the share of all firms that fortify or the share of all supply that is fortified.

Fifth, learning across counties should be promoted. We observed considerable variation across counties in the extent to which they prioritize LSFF and effectively surveil and enforce the mandate. Rather than thinking of training as an activity mostly conducted by the national government for the counties, structures can be created to allow counties to learn about others’ best practices; discuss different ways to handle the program on a tight budget; and benchmark their progress.

Sixth, efforts should continue to reach out to medium-scale and small-scale millers, which are more impacted than their large-scale counterparts by the costs associated with fortification [43]. This suggests that more consideration be given to waiving taxes on equipment and fortificants. Outreach to (and oversight of) small millers should be pursued. Premix suppliers might also be encouraged to make premix available in smaller quantities. Finally, there is an opportunity for large-scale firms to train smaller-scale firms in fortification practices, and such cooperation can be facilitated by development partners.

## 5. Conclusions

In this study, we presented a novel tool for the evaluation of the P2E for LSFF. The framework is comprehensive, straightforward, and applicable at low cost to diverse country settings. It is based on 18 indicators within a tripartite structure that spans the policy agenda, policy implementation, and policy monitoring and evaluation domains of the policy enabling environment. Its application informs on what is working well and where improvements are needed to ensure successful and sustainable LSFF programs.

We then applied the tool to the case of LSFF in Kenya and found that Kenya has achieved the greatest success around policy agenda setting, somewhat less success around policy implementation, and the least success around policy monitoring and evaluation. A validation event held before the results were finalized confirmed that this framework resonates with stakeholders, and there was general agreement on the scores issued for each indicator—even when the score was low.

In the future, this framework should be tested in other settings, including those where the LSFF program is nascent or struggling. The framework should also be applied over time to understand how it can be used to track progress over time and motivate improvement in different P2E domains. The application of the framework to different countries will yield new data that can inform on the similarities and differences in experiences across different country archetypes. As the framework is applied more widely, our understanding of the P2E for LSFF will continue to deepen.

## Supporting information

S1 Text Guiding questions for semi-structured interviews with key informants. (DOCX)

S2 Text Questionnaire to evaluate perceptions of the policy enabling environment for LSFF. (DOCX)

S3 Text Fig A. Food fortification value chain map, Fig B. Perceptions of LSFF policy agenda setting, Fig C. Perceptions of LSFF policy implementation, Fig D. Perceptions of LSFF policy monitoring and evaluation, Table A. Standards of fortification for salt, vegetable oils, maize flour, and wheat flour, Table B. Key informant interviews, Table C. Stakeholder perceptions survey.
(DOCX)

S4 Text Inclusivity in global research. (DOCX)
