# Supplementary material for: Assessment of the policy enabling environment for large-scale food fortification: A novel framework with an application to Kenya
Source: PLOS Glob Public Health. 2024 May 16;4(5):e0003211. doi: 10.1371/journal.pgph.0003211 (PMC11098474; doi:10.1371/journal.pgph.0003211)
Supplement: S1 Text — (DOCX) [file pgph.0003211.s001.docx]

## Supporting information 1 (S1)

## Guiding Questions for Semi-Structured Interviews with Key Informants

The following questions for semi-structured interviews are framed around the LSFF policy enabling environment framework (section 3.1), covering topics related to the policy agenda setting, policy implementation, and policy monitoring/evaluation within a given country. Interviews should be conducted with representatives from government (at the national, regional, and local levels); industry (across the various food vehicles, inclusive of both large and small firms, and inclusive of rural and urban firms); civil society organizations; development partners; and academia. As key informants would be drawn from a diverse set of stakeholders, the following questions would not be asked in each and every interview but would be selectively referenced to guide the conversations.

| **Policy Agenda Setting**  *Policy prioritization*  *Major events*   - Did the country participate/organize any major events on food fortification? - What value does participation in major events, such as a National Food Fortification Summit, bring to the LSFF program?   *Presence of powerful advocates*  *-* Who are the policy “champions” (powerful advocates of LSFF)?   - Who are the veto players that have decision making power within the policy system and vis-à-vis the advocated policy response? Who holds hidden power? - Do policy champions have a strong relationship with the veto players and those with hidden power? If so, is the relationship positive or negative?   *Policy formulation*  *Consultation with stakeholders*   - Was there consultation between policy makers, civil society, and private sector in the design of the LSFF legislations? - Were food processing industries engaged in the process of developing fortification standards? - Was there collaboration among different ministries with responsibility for LSFF?   *Existence of laws and regulations*   - Which laws and regulations exist on LSFF?   *Clarity of legislation*   - Is the legislation on food fortification clear and understandable? If not, what is not clear? - Are the enforcement mechanisms and consequences of noncompliance widely understood? If not, what are the points of confusion? - Do the laws in place clearly present the roles of different stakeholders? If not, what is ambiguous? - Do the laws clearly convey the array of enforcement mechanisms that can be used legally to enforce the fortification mandate? - Do public officers responsible for fortification have clear mandates? If not, what is ambiguous? - Do the fortification mandates apply to all firms (in an even manner) producing the relevant food items? In other words, does the application and enforcement of the law foster a level playing field across the industry, or does it advantage some firms over others? - Are the regulations facilitative or punitive? What can be improved?   *Program meets needs*   - Does the LSFF legislation meet the needs of the population? Why or why not? - Are the national standards appropriate in relation to the micronutrient deficiencies in the population and industry capacity? Why or why not?   **Policy Implementation**  *Stakeholder engagement*  *Sustained consultation*   - Is there sustained consultation among stakeholders in the implementation of the program? - Do all stakeholders understand well how to implement the LSFF program and the need for it? - Are all stakeholders invited to actively participate in meetings and other fora on LSFF?   *Effective coordination*   - Is there a coordination mechanism across different sectors to manage fortification activities? What works well, and what does not work well, when it comes to coordination? - Are all relevant levels of government engaged in this coordination mechanism? - Are industry representatives meaningfully engaged in this coordination mechanism? - Are civil society organizations meaningfully engaged in this coordination mechanism? Are development partners engaged in a meaningful way? - Are the coordinating bodies adequately funded? - How would you characterize coordination between the national and regional/provincial governments when it comes to LSFF implementation?   *Continued support from stakeholders*   - Are stakeholders supportive of the implementation of the LSFF programs? - Is there technical and financial support from regional bodies? - What kind of assistance is provided to the different stakeholders and by whom? - Is there adequate public investment in food fortification in the country? - Is there adequate private sector investment in food fortification in the country.   *Capacities*  *Capacity of industries*   - What do you perceive to be the barriers to compliance with national fortification standards at the industry level? - Are there strong, representative industry or producer associations? - Do these industry associations (producers’ associations or manufacturers’ associations) support fortification, for example by facilitating the joint procurement of equipment and premixes? - Do firms keep records of food fortification, premix supplies and usage, laboratory tests and analysis reports?   *Capacity of regulatory agencies*   - Is there adequate funding at the level of regulatory agencies to implement food fortification? - Is funding for regulatory industries sustainable over the next five years? Is the intervention financially sustainable when donor support diminishes? - Do public officers responsible for fortification have technical and logistical capacity? Is there adequate technical and laboratory capacity in the relevant national government ministries? - Are there enough staff, and have they been trained to undertake industry-level inspection and audits? - Are public laboratories available at the regional or local level to facilitate timely testing and reporting?   *Level of compliance*   - Is the level of industry compliance satisfactory? Why or why not? - How does this differ across products (e.g., salt, oil, wheat flour, maize flour), firm size, geography and/or market concentration?   **Policy Monitoring and Evaluation**  *Oversight and enforcement*  *Guidelines for monitoring*   - Are there guidelines and standardized processes for monitoring fortification? - Are the guidelines clear and easy to understand? - Is there a central database for monitoring? - Is there trust between government inspectors and food processors? Do private sector stakeholders have trust in government officials? - Are there mechanisms for ensuring mutual accountability between governments, donors, the private sector, and citizens? - Are the monitoring processes manageable for firms or unduly burdensome? - Do the relevant industries have their own internal standards for fortification Quality Assurance/Quality Control (QA/QC)?   *Guidelines for enforcement*   - Are there guidelines for enforcement? - Are the guidelines clear and easy to understand? - Among political leaders, are there perceived political risks of enforcement? - Is there bureaucratic autonomy in enforcement?   *Enforcement of standards/regulations*   - Are the standards and regulations enforced (consistently, fairly, transparently)? - Are the penalties in place effective? If not, why do you feel they are not effective?   *Evaluation and reform*  *Existence of assessment dataset*   - Does a database on food fortification volumes and/or compliance rates exist? If yes, how is the data tracked and reported? By whom? How is it utilized? - Does a database on population micronutrient deficiencies exist? If yes, how is the data tracked and reported? By whom? How is it utilized?   *Program reach and effectiveness*   - What is known about the effectiveness of the LSFF program in this country? - What efforts are underway to gather evidence? Are these efforts adequately funded? - Are results taken into consideration by policy makers in terms of policy evaluation and reform?   *Consumer education and awareness*   - To what extent are consumers aware of the importance of LSFF, accept fortified foods, and how to identify fortified products in the market? - What role do civil society organizations play in consumer education and awareness of LSFF? |
| --- |

## 
