# Supplementary material for: Assessment of the policy enabling environment for large-scale food fortification: A novel framework with an application to Kenya
Source: PLOS Glob Public Health. 2024 May 16;4(5):e0003211. doi: 10.1371/journal.pgph.0003211 (PMC11098474; doi:10.1371/journal.pgph.0003211)
Supplement: S2 Text — (DOCX) [file pgph.0003211.s002.docx]

**Supporting information 2 (S2)**

## Questionnaire to Evaluate Perceptions of the Policy Enabling Environment for LSFF

The following questionnaire was developed to elicit stakeholder perceptions of the policy enabling environment for LSFF—information that would feed into a country’s index. The questionnaire is expected to take about 10-15 minutes to complete.

| **Stakeholder Perceptions Survey**  **A. INFORMATION ABOUT YOURSELF AND THE ORGANIZATION YOU REPRESENT**  A1. Name:  A2. Position:  A3. Organization:  A4. Stakeholder group:   \| Government (National level)  Government (County level)  Civil society  Development partner \| Industry  Research/academia  Other: \| \| --- \| --- \|   A4.1 If A4 = “Industry”, please specify the relevant food product(s) with which you work. *Select all that apply.*   \| Maize flour  Wheat flour  Vegetable oils and fats \| Salt  Other: \| \| --- \| --- \|   A5. County/Region of residence:  A6. Is your place of residence rural, peri-urban, or urban?  Rural  Peri-urban  Urban |
| --- | --- | --- | --- | --- |
| **B. GENERAL PERCEPTIONS OF LARGE-SCALE FOOD FORTIFICATION IN THE COUNTRY**  **B1. Policy Agenda Setting**  To what extent do you agree or disagree with the following statements?   \|  \| Completely agree \| Somewhat agree \| Somewhat disagree \| Completely disagree \| Do not know \| \| --- \| --- \| --- \| --- \| --- \| --- \| \| A major event (i.e., crisis, summit) has attracted the attention of the public, industry and/or policy makers to large-scale food fortification. \|  \|  \|  \|  \|  \| \| There are powerful advocates for large-scale food fortification in the country. \|  \|  \|  \|  \|  \| \| There was consultation among stakeholders in the design of the large-scale food fortification legislations. \|  \|  \|  \|  \|  \| \| There exist laws and/or regulations on large-scale food fortification in the country. \|  \|  \|  \|  \|  \| \| The legislation related to large-scale food fortification is clear/ easy to understand. \|  \|  \|  \|  \|  \| \| The large-scale food fortification program is well-designed to meet the population needs in terms of types and amounts of nutrients and choice(s) of food vehicle. \|  \|  \|  \|  \|  \|   B1.1 (Optional) Use this space to clarify any of your responses in B1. |

| **B2. Policy Implementation**  To what extent do you agree or disagree with the following statements?   \|  \| Completely agree \| Somewhat agree \| Somewhat disagree \| Completely disagree \| Do not know \| \| --- \| --- \| --- \| --- \| --- \| --- \| \| There is sustained consultation among stakeholders in the implementation of the FF program (i.e., the program is well communicated and understood). \|  \|  \|  \|  \|  \| \| There is effective coordination among stakeholders in the implementation of the LSFF program (i.e., roles and responsibilities are well defined and complementary). \|  \|  \|  \|  \|  \| \| There is continued support in terms of enthusiasm, engagement, and assistance from the stakeholders in the implementation of the LSFF program. \|  \|  \|  \|  \|  \| \| Industries have adequate financial/human/physical capacity to meet the fortification requirements.  *Industry actors include maize/wheat flour, oil, and salt processors that produce packaged products.* \|  \|  \|  \|  \|  \| \| Regulatory agencies have adequate financial/ human/ physical capacity to monitor and enforce the fortification requirements. \|  \|  \|  \|  \|  \| \| There is a satisfactory level of industry compliance with the fortification requirements. \|  \|  \|  \|  \|  \|   B2.1 (Optional) Use this space to clarify any of your responses in B2. |
| --- | --- | --- | --- | --- | --- | --- | --- | --- | --- | --- | --- | --- | --- | --- | --- | --- | --- | --- | --- | --- | --- | --- | --- | --- | --- | --- | --- | --- | --- | --- | --- | --- | --- | --- | --- | --- | --- | --- | --- | --- | --- | --- |

| **B3. Policy Monitoring and Evaluation**  To what extent do you agree or disagree with the following statements?   \|  \| Completely agree \| Somewhat agree \| Somewhat disagree \| Completely disagree \| Do not know \| \| --- \| --- \| --- \| --- \| --- \| --- \| \| There exist clear guidelines for monitoring of large-scale food fortification. \|  \|  \|  \|  \|  \| \| There exist clear guidelines for enforcement of large-scale food fortification. \|  \|  \|  \|  \|  \| \| The fortification requirements are adequately enforced (i.e., they are enforced consistently, fairly, and transparently). \|  \|  \|  \|  \|  \| \| Data on large-scale food fortification (i.e., volumes/compliance rates) and population micronutrient deficiencies are tracked and reported over time. \|  \|  \|  \|  \|  \| \| Program reach and effectiveness is satisfactory. \|  \|  \|  \|  \|  \| \| Consumers are aware of the importance of fortified foods, accept fortified foods and know how to identify fortified products in the market. \|  \|  \|  \|  \|  \|   B3.1 (Optional) Use this space to clarify any of your responses in B3. |
| --- | --- | --- | --- | --- | --- | --- | --- | --- | --- | --- | --- | --- | --- | --- | --- | --- | --- | --- | --- | --- | --- | --- | --- | --- | --- | --- | --- | --- | --- | --- | --- | --- | --- | --- | --- | --- | --- | --- | --- | --- | --- | --- |
