# Supplementary material for: Assessment of the policy enabling environment for large-scale food fortification: A novel framework with an application to Kenya
Source: PLOS Glob Public Health. 2024 May 16;4(5):e0003211. doi: 10.1371/journal.pgph.0003211 (PMC11098474; doi:10.1371/journal.pgph.0003211)
Supplement: S3 Text — Fig A. Food fortification value chain map, Fig B. Perceptions of LSFF policy agenda setting, Fig C. Perceptions of LSFF policy implementation, Fig D. Perceptions of LSFF policy monitoring and evaluation, Table A. Standards of fortification for salt, vegetable oils, maize flour, and wheat flour, Table B. Key informant interviews, Table C. Stakeholder perceptions survey. (DOCX) [file pgph.0003211.s003.docx]

**Supporting Information 3 (S3 Text)**


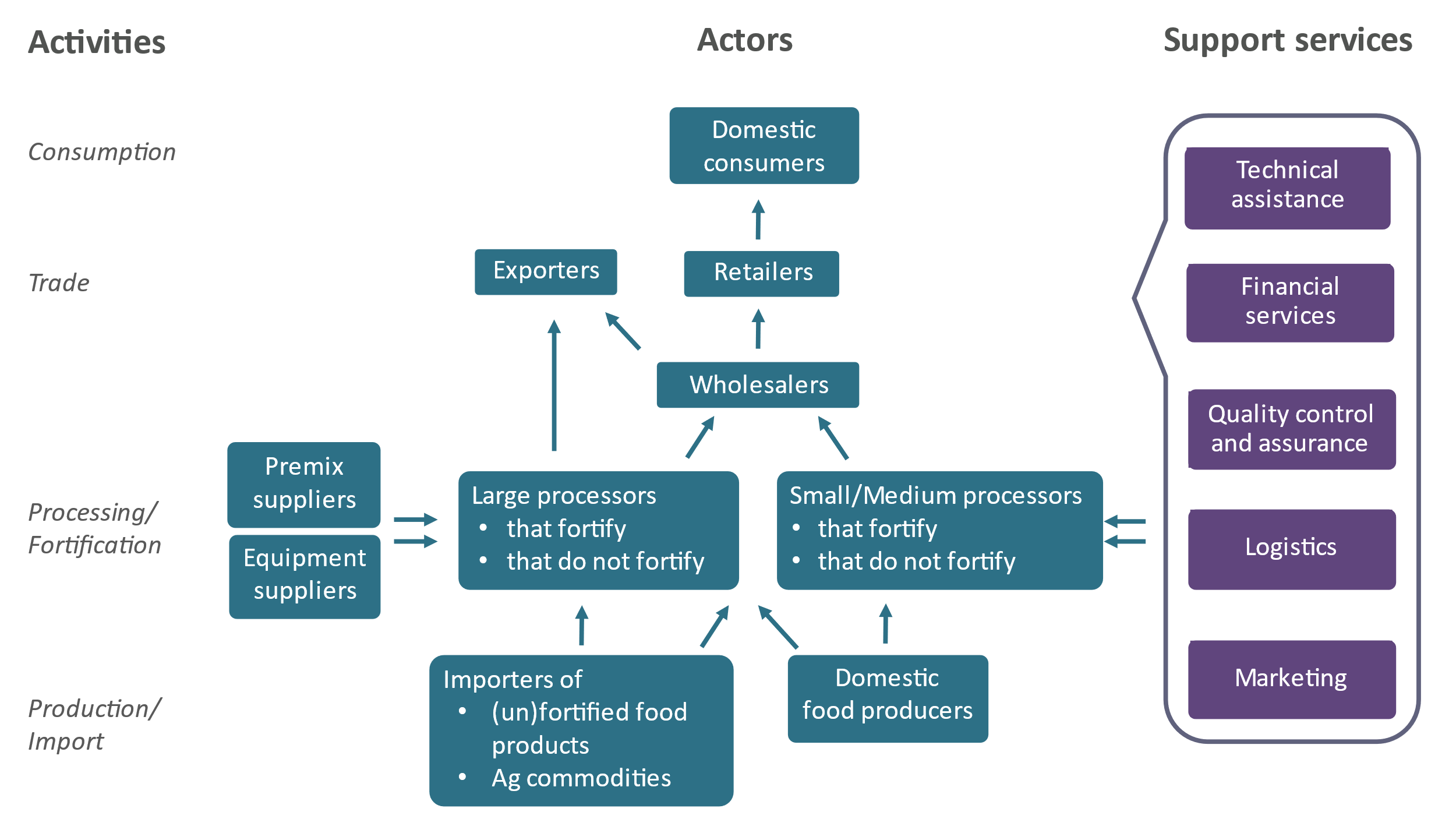


Fig A. Food fortification value chain map

*Source: Authors*

Fig B. Perceptions of LSFF policy agenda setting

*Source: Authors*

Fig C. Perceptions of LSFF policy implementation

*Source: Authors*

Fig D. Perceptions of LSFF policy monitoring and evaluation

*Source: Authors*

Table A. Standards of fortification for salt, vegetable oils, maize flour, and wheat flour

| Product | Fortificants | Year of implementation | Standards for key fortificants |
| --- | --- | --- | --- |
| Salt | Iodine | 1978 | Iodine: 50–84 mg/kg |
| Maize flour | Iron, zinc, folic acid, vitamin B_1,_ B_2_, B_3_ (niacin), B_6,_ B_9,_ and B_12_ and vitamin A | 2012 | Vitamin A: 0.5–1.4mg/kg  Zinc: 33–65mg/kg  Iron: 21–41mg/kg |
| Wheat flour | Iron, zinc, folic acid, vitamin B_1,_ B_2_, B_3_ (niacin), B_6,_ B_9,_ and B_12_ and vitamin A | 2012 | Zinc: 40–80mg/kg  Iron: >20mg/kg |
| Vegetable oils and fats | Vitamin A (retinol) | 2012 | Vitamin A: ~30 mg/kg |

*Source: GoK 2018; Fiedler et al. 2014; Makhumula et al. 2014; Global Fortification Data Exchange 2022*

**Table B. Key informant interviews**

| Stakeholder group | No. of informants |
| --- | --- |
| Government (national) | 2 |
| Government (county) | 6 |
| Industry | 4 |
| Civil society organization | 3 |
| Development partner | 5 |
| Research/Academia | 1 |
| Total | 21 |

*Source: Authors*

Table C. Stakeholder perceptions survey

| Stakeholder group | No. respondents |
| --- | --- |
| Government | 13 |
| Industry | 8 |
| Civil society organization | 2 |
| Development partner | 5 |
| Research/Academia | 16 |
| Other | 2 |
| Total | 46 |

*Source: Authors*
